# {2,2′-[1,1′-(Ethane-1,2-diyldinitrilo)­diethyl­idyne]diphenolato}bis­(pyrrolidine)cobalt(III) perchlorate *p*-xylene hemisolvate

**DOI:** 10.1107/S160053681004660X

**Published:** 2010-11-17

**Authors:** Mehdi Salehi, Grzegorz Dutkiewicz, Maciej Kubicki

**Affiliations:** a Department of Chemistry, Faculty of Science, Semnan University, Semnan, Iran; b Department of Chemistry, Adam Mickiewicz University, Grunwaldzka 6, 60-780 Poznań, Poland

## Abstract

In the mononuclear title complex, [Co(C_18_H_18_N_2_O_2_)(C_4_H_9_N)_2_]ClO_4_·0.5C_8_H_10_, the Co^III^ ion has a slightly distorted octa­hedral coordination geometry. In the Me–salen ligand, the benzene rings are almost parallel, making a dihedral angle of 0.48 (13)°, but the torsion angle along the central C—C bond is 41.1 (2)°·The pyrrolidine rings are in slightly distorted chair conformations. The N atoms of the pyrrolidine axial ligands are involved in N—H⋯O hydrogen bonds with the perchlorate anions, and these hydrogen bonds connect the ionic species into infinite chains along the *b* axis. Some relatively short C—H⋯π inter­actions are also present in the crystal structure and C—H⋯O inter­actions occur. The guest solvent *p*-xylene mol­ecule lies on a special position at the inversion centre.

## Related literature

For the properties of Co(III) complexes with Schiff base ligands, see: Polson *et al.* (1997 ▶); Yamada *et al.* (1999 ▶); Henson *et al.* (1999 ▶); Bianchini & Zoeliner (1997 ▶); Mishra *et al.* (2008 ▶); Kumar *et al.* (2009 ▶). For related structures, see: Dreos *et al.* (2003 ▶). For the preparation of *N*,*N*′-bis­(methyl­salicyl­idene)-1,2-ethyl­enediamine, see: Hariharan & Urbach (1969 ▶). 
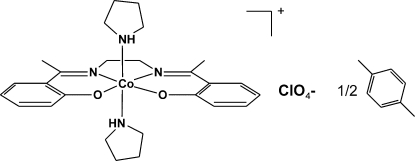


## Experimental

### 

#### Crystal data

[Co(C_18_H_18_N_2_O_2_)(C_4_H_9_N)_2_]ClO_4_·0.5C_8_H_10_
*M* *_r_* = 648.05
Monoclinic, 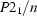

*a* = 13.118 (2) Å
*b* = 16.551 (3) Å
*c* = 13.784 (2) Å
β = 92.87 (2)°
*V* = 2989.0 (8) Å^3^
*Z* = 4
Mo *K*α radiation
μ = 0.71 mm^−1^
*T* = 100 K
0.40 × 0.15 × 0.15 mm

#### Data collection

Oxford Diffraction Xcalibur Eos diffractometer
Absorption correction: multi-scan (*CrysAlis PRO*; Oxford Diffraction, 2009 ▶) *T*_min_ = 0.422, *T*_max_ = 1.000
23760 measured reflections
7081 independent reflections
4734 reflections with *I* > 2σ(*I*)
*R*_int_ = 0.053

#### Refinement

*R*[*F* ^2^ > 2σ(*F* ^2^)] = 0.040
*wR*(*F* ^2^) = 0.084
*S* = 0.99
7081 reflections
502 parameters
H atoms treated by a mixture of independent and constrained refinement
Δρ_max_ = 0.67 e Å^−3^
Δρ_min_ = −0.51 e Å^−3^

### 

Data collection: *CrysAlis PRO* (Oxford Diffraction, 2009 ▶); cell refinement: *CrysAlis PRO*; data reduction: *CrysAlis PRO*; program(s) used to solve structure: *SIR92* (Altomare *et al.*, 1993 ▶); program(s) used to refine structure: *SHELXL97* (Sheldrick, 2008 ▶); molecular graphics: *Stereochemical Workstation Operation Manual* (Siemens, 1989 ▶); software used to prepare material for publication: *SHELXL97*.

## Supplementary Material

Crystal structure: contains datablocks I, global. DOI: 10.1107/S160053681004660X/jh2227sup1.cif

Structure factors: contains datablocks I. DOI: 10.1107/S160053681004660X/jh2227Isup2.hkl

Additional supplementary materials:  crystallographic information; 3D view; checkCIF report

## Figures and Tables

**Table 1:** Hydrogen-bond geometry (Å, °) *CgA*, *CgB* and *CgC* are the centroids of the C11–C17, C26–C32 and C1*A*–C3*A*,C1*A*′–C3*A*′ rings, respectively.

*D*—H⋯*A*	*D*—H	H⋯*A*	*D*⋯*A*	*D*—H⋯*A*
N1—H1⋯O4*A*^i^	0.90 (2)	2.32 (2)	3.200 (2)	166.2 (19)
N6—H6⋯O2*A*	0.89 (2)	2.55 (2)	3.244 (2)	135.9 (18)
N6—H6⋯O3*A*	0.89 (2)	2.33 (2)	3.181 (3)	160.9 (18)
C2*A*—H2*A*⋯O2*A*	0.88 (2)	2.61 (3)	3.477 (3)	166 (2)
C19—H19*B*⋯*CgA*^ii^	0.92 (2)	2.85 (2)	3.438 (2)	122.8 (16)
C14—H14⋯*CgB*^iii^	0.94 (2)	2.56 (2)	3.430 (2)	153.8 (17)
C22—H222⋯*CgC*^iv^	1.00 (2)	2.92 (2)	3.784 (2)	145.5 (16)

Symmetry codes: (i) 
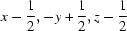
; (ii) 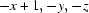; (iii) 
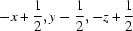
; (iv) 
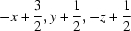
.

**Fig. 1.**
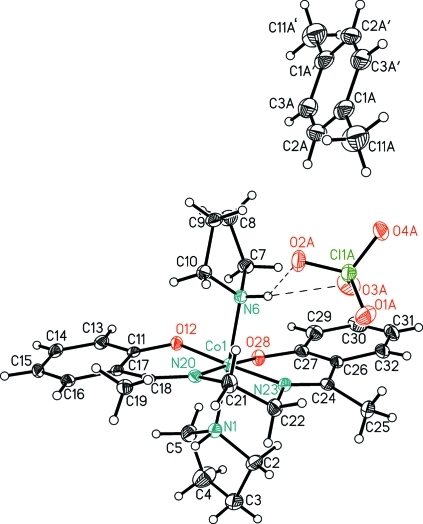
The perspective view of the cation (I), with atom labeling scheme. Displacement ellipsoids are drawn at 50% probability level, hydrogen atoms are depicted as spheres of arbitrary radii. Hydrogen bonds are shown as dashed lines, prime signs refer to the symmetry code 2 - x,-y,1 - z.

**Fig. 2.**
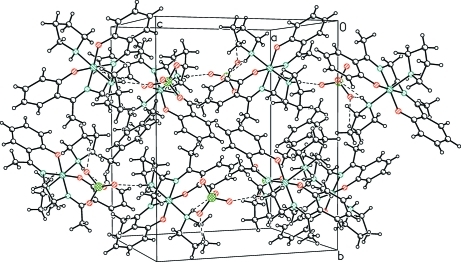
The crystal packing of (I).The dashed lines denote hydrogen bonds.

## supplementary crystallographic information

### Comment

Cobalt Schiff base complexes with tetradentate Schiff base ligands have been extensively used to mimic cobalamin (B12) coenzymes (Polson *et al.*, 1997), dioxygen carriers and oxygen activators (Yamada *et al.*, 1999, Henson *et al.*, 1999, Bianchini & Zoeliner, 1997). Co(III) Schiff base complexes with two amines in axial positions have been used as antimicrobial agents as well (Mishra *et al.*, 2008, Kumar *et al.*, 2009). Here we present the structure of Co[Me-salen](bis-pyrrolidine) which crystallizes as the *p*-xylene solvate, (I, Scheme 1).

Fig. 1 shows the perspective view of the cation. The Co atom is six-coordinated by two N atoms and two O atoms from Schiff base ligand, which are coplanar within 0.048 (1)Å and two N atoms from two pyrrolidine molecules, which are *trans* to each other (N—Co—N angle is 176.75 (8)°). These two axial ligands, both in half-chair conformations, are rotated by about 90° with respect to each other. The small distortion from the ideal octahedral coordination can be seen in the deviations of the *trans* angles from the ideal values of 180° as well as in the angle between the least-squares planes through six-membered chelate rings (Co—O—C—C—C—N—Co) of 1.82 (9)°. The Co—O and Co—N bond lengths agree well with those found in analogous cobalt complexes (*e.g.*, Dreos *et al.*, 2003).

In the crystal structure, the N atoms of the pyrrolidine ligands are involved in N—H···O hydrogen bonds with the perchlorate anions. These hydrogen bonds connect molecules into infinite chains along the *b* axis (Fig. 2). Some C—H···π interactions (Table 2) also may play some role in determining the crystal structure. Especially one of these contacts (C14—H14···CgB (1/2 - *x*,-1/2 + *y*,1/2 - *z*) might be regarded as weak hydrogen bond.

### Experimental

The desired ligand, H_2_Me-salen, was synthesized according to the literature procedures (Hariharan & Urbach, 1969). To a stirring solution of Co(CH_3_COO)_2_.4H_2_O (0.125 g, 0.5 mmol) in methanol (25 ml) was added an equimolar of H_2_Me-salen (0.148 g, 0.5 mmol). The red solution turned brown immediately upon the formation of [Co^II^(Me-salen)] complex. To this solution was added 4 mmol of pyrrolidine, and air was bubbled through the reaction mixture for about 3 h. 0.5 mmol (0.0615 g) of NaClO_4 _was then added to the resulting brown solution and stirred for 5 minutes. A red microcrystalline solid was produced by slow evaporation of methanol at room temperature. The product was then recrystallized from chloroform-xylene (2:1 *v*/*v*) and dark red crystals suitable for X-ray crystallography were obtained. The crystals were filtered off and washed with a small amount of cold methanol and dried under vacuum. Yield: 55%..

### Refinement

The positions of H atoms were freely refined, and their *U*_iso_ values were set at 1.2 (1.5 for methyl groups) times *U*_eq_ of appropriate carrier atom.

### Crystal data

**Table:**

[Co(C_18_H_18_N_2_O_2_)(C_4_H_9_N)_2_]ClO_4_·0.5C_8_H_10_	*F*(000) = 1364
*M**_r_* = 648.05	*D*_x_ = 1.440 Mg m^−^^3^
Monoclinic, *P*2_1_/*n*	Mo *K*α radiation, λ = 0.71073 Å
Hall symbol: -P 2yn	Cell parameters from 9456 reflections
*a* = 13.118 (2) Å	θ = 2.9–29.0°
*b* = 16.551 (3) Å	µ = 0.71 mm^−^^1^
*c* = 13.784 (2) Å	*T* = 100 K
β = 92.87 (2)°	Prism, dark red
*V* = 2989.0 (8) Å^3^	0.4 × 0.15 × 0.15 mm
*Z* = 4	

### Data collection

**Table:**

Oxford Diffraction Xcalibur Eos diffractometer	7081 independent reflections
Radiation source: Enhance (Mo) X-ray Source	4734 reflections with *I* > 2σ(*I*)
graphite	*R*_int_ = 0.053
Detector resolution: 16.1544 pixels mm^-1^	θ_max_ = 29.1°, θ_min_ = 2.9°
ω scans	*h* = −16→15
Absorption correction: multi-scan (*CrysAlis PRO*; Oxford Diffraction, 2009)	*k* = −21→22
*T*_min_ = 0.422, *T*_max_ = 1.000	*l* = −18→18
23760 measured reflections	

### Refinement

**Table:**

Refinement on *F*^2^	Primary atom site location: structure-invariant direct methods
Least-squares matrix: full	Secondary atom site location: difference Fourier map
*R*[*F*^2^ > 2σ(*F*^2^)] = 0.040	Hydrogen site location: inferred from neighbouring sites
*wR*(*F*^2^) = 0.084	H atoms treated by a mixture of independent and constrained refinement
*S* = 0.99	*w* = 1/[σ^2^(*F*_o_^2^) + (0.038*P*)^2^] where *P* = (*F*_o_^2^ + 2*F*_c_^2^)/3
7081 reflections	(Δ/σ)_max_ = 0.002
502 parameters	Δρ_max_ = 0.67 e Å^−^^3^
0 restraints	Δρ_min_ = −0.51 e Å^−^^3^

### Special details

**Table:**

Geometry. All s.u.'s (except the s.u. in the dihedral angle between two l.s. planes) are estimated using the full covariance matrix. The cell s.u.'s are taken into account individually in the estimation of s.u.'s in distances, angles and torsion angles; correlations between s.u.'s in cell parameters are only used when they are defined by crystal symmetry. An approximate (isotropic) treatment of cell s.u.'s is used for estimating s.u.'s involving l.s. planes.
Refinement. Refinement of *F*^2^ against ALL reflections. The weighted *R*-factor *wR* and goodness of fit *S* are based on *F*^2^, conventional *R*-factors *R* are based on *F*, with *F* set to zero for negative *F*^2^. The threshold expression of *F*^2^ > 2σ(*F*^2^) is used only for calculating *R*-factors(gt) *etc*. and is not relevant to the choice of reflections for refinement. *R*-factors based on *F*^2^ are statistically about twice as large as those based on *F*, and *R*- factors based on ALL data will be even larger.

### Fractional atomic coordinates and isotropic or equivalent isotropic displacement parameters (Å^2^)

**Table:**

	*x*	*y*	*z*	*U*_iso_*/*U*_eq_	
Co1	0.43307 (2)	0.200878 (17)	0.223086 (19)	0.01430 (8)	
N1	0.32241 (13)	0.26626 (11)	0.15288 (13)	0.0195 (4)	
H1	0.3269 (16)	0.2648 (13)	0.0877 (17)	0.023*	
C2	0.31534 (19)	0.35365 (14)	0.17595 (19)	0.0280 (6)	
H21	0.3731 (18)	0.3871 (15)	0.1487 (16)	0.034*	
H22	0.3120 (17)	0.3579 (14)	0.2515 (17)	0.034*	
C3	0.21166 (19)	0.37890 (16)	0.1324 (2)	0.0317 (6)	
H31	0.1857 (17)	0.4294 (16)	0.1617 (17)	0.038*	
H32	0.2132 (18)	0.3776 (15)	0.0624 (18)	0.038*	
C4	0.1450 (2)	0.30912 (17)	0.1558 (3)	0.0427 (7)	
H41	0.085 (2)	0.3023 (16)	0.107 (2)	0.051*	
H42	0.121 (2)	0.3201 (17)	0.218 (2)	0.051*	
C5	0.21586 (18)	0.23502 (15)	0.16164 (19)	0.0262 (5)	
H51	0.2149 (17)	0.2099 (14)	0.2271 (17)	0.031*	
H52	0.1983 (18)	0.1966 (15)	0.1083 (17)	0.031*	
N6	0.53743 (14)	0.13130 (11)	0.29580 (11)	0.0160 (4)	
H6	0.5884 (16)	0.1644 (14)	0.3137 (15)	0.019*	
C7	0.50197 (19)	0.09102 (14)	0.38566 (15)	0.0207 (5)	
H71	0.4302 (17)	0.0819 (14)	0.3787 (15)	0.025*	
H72	0.5138 (16)	0.1294 (14)	0.4397 (15)	0.025*	
C8	0.5615 (2)	0.01142 (15)	0.39679 (17)	0.0260 (5)	
H81	0.5990 (17)	0.0093 (14)	0.4590 (17)	0.031*	
H82	0.5160 (17)	−0.0334 (15)	0.3918 (16)	0.031*	
C9	0.63361 (18)	0.00956 (15)	0.31396 (16)	0.0211 (5)	
H91	0.6997 (17)	0.0345 (13)	0.3372 (15)	0.025*	
H92	0.6430 (17)	−0.0387 (14)	0.2914 (15)	0.025*	
C10	0.58171 (18)	0.06470 (14)	0.23889 (15)	0.0189 (5)	
H101	0.6284 (16)	0.0877 (13)	0.1909 (15)	0.023*	
H102	0.5226 (16)	0.0385 (13)	0.2052 (15)	0.023*	
C11	0.33034 (15)	0.06975 (12)	0.12952 (14)	0.0145 (4)	
O12	0.35375 (10)	0.10722 (8)	0.21165 (9)	0.0160 (3)	
C13	0.25165 (17)	0.01153 (13)	0.13147 (16)	0.0188 (5)	
H13	0.2275 (16)	0.0008 (14)	0.1890 (16)	0.023*	
C14	0.21879 (17)	−0.03030 (13)	0.05014 (16)	0.0199 (5)	
H14	0.1637 (16)	−0.0666 (14)	0.0540 (15)	0.024*	
C15	0.26181 (16)	−0.01498 (13)	−0.03840 (15)	0.0183 (5)	
H15	0.2360 (15)	−0.0463 (13)	−0.0936 (15)	0.022*	
C16	0.33976 (16)	0.04004 (13)	−0.04243 (15)	0.0163 (5)	
H16	0.3683 (15)	0.0523 (13)	−0.1003 (15)	0.020*	
C17	0.37838 (15)	0.08255 (12)	0.04054 (14)	0.0139 (4)	
C18	0.46758 (15)	0.13478 (12)	0.03271 (13)	0.0139 (4)	
C19	0.52757 (18)	0.12788 (14)	−0.05752 (15)	0.0181 (5)	
H19A	0.4929 (17)	0.1555 (14)	−0.1079 (16)	0.027*	
H19B	0.5293 (16)	0.0748 (15)	−0.0782 (15)	0.027*	
H19C	0.5991 (18)	0.1489 (14)	−0.0482 (15)	0.027*	
N20	0.49586 (13)	0.18431 (10)	0.10260 (11)	0.0152 (4)	
C21	0.58643 (18)	0.23621 (14)	0.09577 (16)	0.0222 (5)	
H211	0.5974 (16)	0.2496 (13)	0.0285 (16)	0.027*	
H212	0.6468 (18)	0.2055 (14)	0.1226 (16)	0.027*	
C22	0.56754 (19)	0.31395 (14)	0.14930 (16)	0.0225 (5)	
H221	0.6309 (18)	0.3398 (14)	0.1644 (15)	0.027*	
H222	0.5206 (17)	0.3489 (14)	0.1087 (16)	0.027*	
N23	0.51726 (13)	0.29409 (10)	0.23916 (12)	0.0166 (4)	
C24	0.52723 (16)	0.34161 (13)	0.31422 (15)	0.0188 (5)	
C25	0.5844 (2)	0.41992 (15)	0.30703 (19)	0.0263 (6)	
H25A	0.652 (2)	0.4135 (16)	0.3180 (17)	0.040*	
H25B	0.5622 (18)	0.4579 (16)	0.3546 (18)	0.040*	
H25C	0.5750 (18)	0.4459 (16)	0.2461 (18)	0.040*	
C26	0.48102 (16)	0.32171 (13)	0.40501 (15)	0.0183 (5)	
C27	0.40293 (16)	0.26336 (13)	0.41079 (14)	0.0186 (5)	
O28	0.36521 (10)	0.22025 (8)	0.33713 (10)	0.0181 (3)	
C29	0.35918 (18)	0.25124 (14)	0.50123 (16)	0.0243 (5)	
H29	0.3078 (18)	0.2120 (14)	0.5022 (16)	0.029*	
C30	0.3937 (2)	0.29266 (16)	0.58313 (17)	0.0293 (6)	
H30	0.3643 (18)	0.2825 (15)	0.6370 (17)	0.035*	
C31	0.47268 (19)	0.34752 (16)	0.57808 (17)	0.0295 (6)	
H31A	0.4954 (18)	0.3735 (15)	0.6275 (17)	0.035*	
C32	0.51534 (19)	0.36198 (14)	0.49171 (17)	0.0253 (5)	
H32A	0.5689 (18)	0.3976 (14)	0.4914 (16)	0.030*	
Cl1A	0.79066 (4)	0.23619 (3)	0.35925 (4)	0.02212 (13)	
O1A	0.80829 (13)	0.30755 (10)	0.30347 (11)	0.0339 (4)	
O2A	0.78027 (12)	0.16794 (9)	0.29433 (10)	0.0280 (4)	
O3A	0.69829 (12)	0.24462 (11)	0.41007 (12)	0.0357 (4)	
O4A	0.87471 (11)	0.22283 (9)	0.42896 (11)	0.0258 (4)	
C1A	1.0458 (2)	0.05050 (15)	0.43188 (17)	0.0345 (6)	
C11A	1.0945 (3)	0.1044 (2)	0.3592 (2)	0.0499 (8)	
H11A	1.171 (2)	0.1142 (19)	0.381 (2)	0.075*	
H11B	1.062 (2)	0.152 (2)	0.349 (2)	0.075*	
H11C	1.097 (2)	0.080 (2)	0.303 (2)	0.075*	
C2A	0.9406 (2)	0.03841 (16)	0.42930 (18)	0.0353 (7)	
H2A	0.9032 (19)	0.0658 (16)	0.3855 (18)	0.042*	
C3A	0.8953 (2)	−0.01037 (16)	0.49598 (18)	0.0360 (6)	
H3A	0.821 (2)	−0.0225 (17)	0.4959 (19)	0.054*	

### Atomic displacement parameters (Å^2^)

**Table:**

	*U*^11^	*U*^22^	*U*^33^	*U*^12^	*U*^13^	*U*^23^
Co1	0.01606 (16)	0.01228 (14)	0.01440 (14)	−0.00159 (13)	−0.00085 (10)	−0.00040 (12)
N1	0.0193 (10)	0.0172 (9)	0.0218 (9)	−0.0016 (8)	−0.0013 (8)	−0.0006 (8)
C2	0.0290 (15)	0.0167 (12)	0.0376 (14)	0.0002 (11)	−0.0065 (11)	0.0018 (11)
C3	0.0296 (15)	0.0254 (14)	0.0390 (15)	0.0056 (12)	−0.0088 (12)	−0.0012 (12)
C4	0.0247 (16)	0.0319 (16)	0.071 (2)	0.0057 (13)	0.0034 (14)	0.0010 (15)
C5	0.0206 (13)	0.0230 (13)	0.0345 (14)	−0.0013 (11)	−0.0027 (10)	−0.0011 (11)
N6	0.0192 (10)	0.0148 (9)	0.0139 (8)	−0.0019 (8)	0.0011 (7)	0.0001 (7)
C7	0.0265 (13)	0.0202 (12)	0.0155 (11)	0.0007 (10)	0.0025 (9)	0.0029 (9)
C8	0.0314 (15)	0.0224 (13)	0.0244 (12)	0.0030 (11)	0.0029 (11)	0.0035 (10)
C9	0.0213 (13)	0.0178 (12)	0.0241 (12)	0.0052 (10)	0.0005 (9)	0.0011 (10)
C10	0.0207 (13)	0.0190 (12)	0.0172 (11)	0.0033 (10)	0.0020 (9)	−0.0022 (9)
C11	0.0145 (11)	0.0108 (10)	0.0180 (10)	0.0025 (9)	−0.0027 (8)	0.0010 (8)
O12	0.0188 (8)	0.0143 (7)	0.0146 (7)	−0.0036 (6)	−0.0005 (6)	−0.0003 (6)
C13	0.0217 (13)	0.0143 (11)	0.0206 (11)	−0.0019 (10)	0.0025 (9)	0.0020 (9)
C14	0.0150 (12)	0.0133 (11)	0.0308 (12)	−0.0016 (9)	−0.0036 (10)	0.0000 (9)
C15	0.0167 (12)	0.0163 (11)	0.0212 (11)	0.0025 (9)	−0.0076 (9)	−0.0046 (9)
C16	0.0183 (12)	0.0155 (11)	0.0149 (10)	0.0052 (9)	−0.0009 (9)	−0.0002 (9)
C17	0.0131 (11)	0.0109 (10)	0.0175 (10)	0.0011 (9)	−0.0031 (8)	0.0014 (8)
C18	0.0146 (11)	0.0135 (10)	0.0132 (10)	0.0034 (9)	−0.0028 (8)	0.0042 (8)
C19	0.0176 (13)	0.0182 (12)	0.0184 (11)	0.0011 (10)	0.0017 (9)	0.0003 (9)
N20	0.0155 (10)	0.0141 (9)	0.0158 (8)	−0.0024 (7)	−0.0008 (7)	0.0034 (7)
C21	0.0238 (13)	0.0233 (12)	0.0198 (11)	−0.0090 (11)	0.0030 (9)	0.0006 (10)
C22	0.0234 (13)	0.0208 (13)	0.0230 (11)	−0.0091 (10)	−0.0021 (10)	0.0023 (9)
N23	0.0153 (10)	0.0142 (9)	0.0201 (9)	−0.0014 (8)	−0.0024 (7)	0.0007 (8)
C24	0.0134 (12)	0.0159 (11)	0.0262 (12)	0.0032 (9)	−0.0089 (9)	0.0000 (9)
C25	0.0278 (15)	0.0174 (12)	0.0328 (14)	−0.0028 (11)	−0.0080 (11)	−0.0037 (11)
C26	0.0168 (12)	0.0163 (11)	0.0211 (11)	0.0068 (9)	−0.0069 (9)	−0.0041 (9)
C27	0.0175 (12)	0.0192 (11)	0.0186 (10)	0.0094 (10)	−0.0040 (9)	−0.0037 (9)
O28	0.0168 (8)	0.0197 (8)	0.0182 (7)	−0.0001 (6)	0.0032 (6)	−0.0042 (6)
C29	0.0229 (13)	0.0258 (14)	0.0245 (12)	0.0059 (11)	0.0023 (10)	−0.0006 (10)
C30	0.0327 (15)	0.0355 (15)	0.0197 (11)	0.0161 (13)	0.0018 (10)	−0.0028 (11)
C31	0.0308 (15)	0.0316 (15)	0.0250 (13)	0.0136 (12)	−0.0109 (11)	−0.0123 (11)
C32	0.0253 (14)	0.0215 (13)	0.0279 (12)	0.0085 (11)	−0.0110 (10)	−0.0060 (10)
Cl1A	0.0213 (3)	0.0208 (3)	0.0241 (3)	−0.0017 (2)	−0.0004 (2)	−0.0035 (2)
O1A	0.0455 (11)	0.0222 (9)	0.0339 (9)	−0.0021 (8)	−0.0005 (8)	0.0039 (8)
O2A	0.0351 (10)	0.0232 (9)	0.0255 (8)	−0.0091 (8)	−0.0027 (7)	−0.0060 (7)
O3A	0.0208 (9)	0.0498 (12)	0.0366 (10)	0.0078 (9)	0.0044 (7)	−0.0034 (9)
O4A	0.0199 (9)	0.0268 (9)	0.0300 (9)	0.0000 (7)	−0.0069 (7)	−0.0057 (7)
C1A	0.0544 (19)	0.0251 (14)	0.0238 (12)	0.0132 (13)	−0.0004 (12)	−0.0075 (11)
C11A	0.072 (2)	0.0429 (19)	0.0357 (16)	0.0097 (17)	0.0069 (16)	−0.0021 (14)
C2A	0.050 (2)	0.0306 (15)	0.0241 (13)	0.0174 (13)	−0.0091 (12)	−0.0067 (11)
C3A	0.0466 (17)	0.0316 (15)	0.0293 (14)	0.0122 (14)	−0.0020 (12)	−0.0100 (12)

### Geometric parameters (Å, °)

**Table:**

Co1—O12	1.8692 (14)	C17—C18	1.463 (3)
Co1—O28	1.8725 (14)	C18—N20	1.305 (2)
Co1—N23	1.9038 (17)	C18—C19	1.509 (3)
Co1—N20	1.9102 (17)	C19—H19A	0.93 (2)
Co1—N6	2.0168 (17)	C19—H19B	0.92 (2)
Co1—N1	2.0184 (18)	C19—H19C	1.00 (2)
N1—C2	1.485 (3)	N20—C21	1.473 (3)
N1—C5	1.501 (3)	C21—C22	1.510 (3)
N1—H1	0.90 (2)	C21—H211	0.97 (2)
C2—C3	1.518 (3)	C21—H212	1.00 (2)
C2—H21	1.02 (2)	C22—N23	1.469 (3)
C2—H22	1.05 (2)	C22—H221	0.95 (2)
C3—C4	1.494 (4)	C22—H222	1.00 (2)
C3—H31	1.00 (3)	N23—C24	1.301 (3)
C3—H32	0.97 (2)	C24—C26	1.455 (3)
C4—C5	1.538 (4)	C24—C25	1.503 (3)
C4—H41	1.01 (3)	C25—H25A	0.89 (3)
C4—H42	0.94 (3)	C25—H25B	0.96 (3)
C5—H51	0.99 (2)	C25—H25C	0.95 (3)
C5—H52	0.99 (2)	C26—C27	1.413 (3)
N6—C10	1.488 (3)	C26—C32	1.422 (3)
N6—C7	1.501 (3)	C27—O28	1.317 (2)
N6—H6	0.89 (2)	C27—C29	1.413 (3)
C7—C8	1.535 (3)	C29—C30	1.378 (3)
C7—H71	0.95 (2)	C29—H29	0.94 (2)
C7—H72	0.99 (2)	C30—C31	1.382 (4)
C8—C9	1.519 (3)	C30—H30	0.87 (2)
C8—H81	0.97 (2)	C31—C32	1.362 (3)
C8—H82	0.95 (2)	C31—H31A	0.85 (2)
C9—C10	1.515 (3)	C32—H32A	0.92 (2)
C9—H91	1.00 (2)	Cl1A—O1A	1.4343 (16)
C9—H92	0.87 (2)	Cl1A—O3A	1.4365 (16)
C10—H101	1.00 (2)	Cl1A—O4A	1.4428 (15)
C10—H102	0.98 (2)	Cl1A—O2A	1.4433 (15)
C11—O12	1.314 (2)	C1A—C2A	1.393 (4)
C11—C13	1.413 (3)	C1A—C3A^i^	1.396 (3)
C11—C17	1.423 (3)	C1A—C11A	1.507 (4)
C13—C14	1.369 (3)	C11A—H11A	1.05 (3)
C13—H13	0.89 (2)	C11A—H11B	0.90 (3)
C14—C15	1.393 (3)	C11A—H11C	0.88 (3)
C14—H14	0.94 (2)	C2A—C3A	1.379 (4)
C15—C16	1.372 (3)	C2A—H2A	0.88 (2)
C15—H15	0.97 (2)	C3A—C1A^i^	1.396 (3)
C16—C17	1.415 (3)	C3A—H3A	1.00 (3)
C16—H16	0.92 (2)		
			
O12—Co1—O28	85.80 (6)	C16—C15—C14	119.4 (2)
O12—Co1—N23	177.53 (7)	C16—C15—H15	123.8 (13)
O28—Co1—N23	93.50 (7)	C14—C15—H15	116.7 (13)
O12—Co1—N20	94.02 (7)	C15—C16—C17	122.3 (2)
O28—Co1—N20	176.62 (7)	C15—C16—H16	121.2 (13)
N23—Co1—N20	86.82 (7)	C17—C16—H16	116.5 (13)
O12—Co1—N6	85.96 (7)	C16—C17—C11	117.82 (18)
O28—Co1—N6	91.14 (6)	C16—C17—C18	119.31 (18)
N23—Co1—N6	91.69 (7)	C11—C17—C18	122.82 (17)
N20—Co1—N6	92.21 (7)	N20—C18—C17	121.05 (18)
O12—Co1—N1	91.16 (7)	N20—C18—C19	121.02 (19)
O28—Co1—N1	87.13 (7)	C17—C18—C19	117.91 (18)
N23—Co1—N1	91.17 (7)	C18—C19—H19A	108.8 (14)
N20—Co1—N1	89.50 (7)	C18—C19—H19B	110.4 (14)
N6—Co1—N1	176.75 (8)	H19A—C19—H19B	104.8 (18)
C2—N1—C5	104.47 (18)	C18—C19—H19C	113.0 (12)
C2—N1—Co1	118.08 (14)	H19A—C19—H19C	110.1 (19)
C5—N1—Co1	115.28 (14)	H19B—C19—H19C	109.4 (19)
C2—N1—H1	104.3 (15)	C18—N20—C21	121.32 (17)
C5—N1—H1	100.2 (14)	C18—N20—Co1	127.82 (14)
Co1—N1—H1	112.4 (14)	C21—N20—Co1	110.86 (13)
N1—C2—C3	104.35 (19)	N20—C21—C22	108.25 (19)
N1—C2—H21	113.1 (13)	N20—C21—H211	110.6 (13)
C3—C2—H21	111.7 (13)	C22—C21—H211	107.9 (13)
N1—C2—H22	106.5 (13)	N20—C21—H212	107.9 (13)
C3—C2—H22	107.1 (12)	C22—C21—H212	113.6 (13)
H21—C2—H22	113.4 (18)	H211—C21—H212	108.5 (18)
C4—C3—C2	102.9 (2)	N23—C22—C21	108.23 (18)
C4—C3—H31	110.2 (14)	N23—C22—H221	110.0 (13)
C2—C3—H31	112.9 (13)	C21—C22—H221	109.1 (14)
C4—C3—H32	103.9 (15)	N23—C22—H222	108.2 (13)
C2—C3—H32	109.1 (14)	C21—C22—H222	109.3 (13)
H31—C3—H32	117 (2)	H221—C22—H222	111.9 (19)
C3—C4—C5	105.6 (2)	C24—N23—C22	120.24 (18)
C3—C4—H41	112.7 (16)	C24—N23—Co1	128.10 (15)
C5—C4—H41	113.1 (16)	C22—N23—Co1	111.44 (13)
C3—C4—H42	105.7 (18)	N23—C24—C26	121.16 (19)
C5—C4—H42	109.4 (18)	N23—C24—C25	120.0 (2)
H41—C4—H42	110 (2)	C26—C24—C25	118.8 (2)
N1—C5—C4	106.5 (2)	C24—C25—H25A	112.2 (17)
N1—C5—H51	105.7 (13)	C24—C25—H25B	110.4 (15)
C4—C5—H51	110.2 (14)	H25A—C25—H25B	107 (2)
N1—C5—H52	110.1 (14)	C24—C25—H25C	114.0 (15)
C4—C5—H52	110.9 (14)	H25A—C25—H25C	107 (2)
H51—C5—H52	113.1 (19)	H25B—C25—H25C	106 (2)
C10—N6—C7	104.56 (16)	C27—C26—C32	118.2 (2)
C10—N6—Co1	115.74 (12)	C27—C26—C24	122.51 (18)
C7—N6—Co1	115.66 (14)	C32—C26—C24	119.3 (2)
C10—N6—H6	107.1 (14)	O28—C27—C29	116.7 (2)
C7—N6—H6	107.8 (14)	O28—C27—C26	124.91 (19)
Co1—N6—H6	105.5 (14)	C29—C27—C26	118.33 (19)
N6—C7—C8	106.65 (18)	C27—O28—Co1	124.32 (13)
N6—C7—H71	109.4 (13)	C30—C29—C27	121.4 (2)
C8—C7—H71	111.7 (14)	C30—C29—H29	122.7 (14)
N6—C7—H72	107.1 (13)	C27—C29—H29	115.9 (14)
C8—C7—H72	114.7 (12)	C29—C30—C31	120.1 (2)
H71—C7—H72	107.2 (18)	C29—C30—H30	117.5 (17)
C9—C8—C7	105.97 (19)	C31—C30—H30	122.4 (17)
C9—C8—H81	110.9 (14)	C32—C31—C30	120.1 (2)
C7—C8—H81	110.7 (14)	C32—C31—H31A	118.0 (17)
C9—C8—H82	110.0 (14)	C30—C31—H31A	121.9 (17)
C7—C8—H82	110.3 (14)	C31—C32—C26	121.7 (2)
H81—C8—H82	108.9 (19)	C31—C32—H32A	117.7 (14)
C10—C9—C8	103.04 (18)	C26—C32—H32A	120.5 (14)
C10—C9—H91	108.8 (13)	O1A—Cl1A—O3A	110.12 (11)
C8—C9—H91	108.3 (12)	O1A—Cl1A—O4A	110.17 (10)
C10—C9—H92	112.1 (15)	O3A—Cl1A—O4A	108.98 (9)
C8—C9—H92	113.1 (15)	O1A—Cl1A—O2A	108.95 (9)
H91—C9—H92	111.1 (19)	O3A—Cl1A—O2A	108.82 (10)
N6—C10—C9	105.08 (16)	O4A—Cl1A—O2A	109.78 (10)
N6—C10—H101	109.6 (13)	C2A—C1A—C3A^i^	117.5 (2)
C9—C10—H101	114.3 (12)	C2A—C1A—C11A	121.3 (2)
N6—C10—H102	104.8 (13)	C3A^i^—C1A—C11A	121.1 (3)
C9—C10—H102	111.9 (13)	C1A—C11A—H11A	109.4 (17)
H101—C10—H102	110.5 (17)	C1A—C11A—H11B	114 (2)
O12—C11—C13	116.42 (18)	H11A—C11A—H11B	111 (3)
O12—C11—C17	125.31 (18)	C1A—C11A—H11C	111 (2)
C13—C11—C17	118.26 (18)	H11A—C11A—H11C	104 (3)
C11—O12—Co1	124.71 (12)	H11B—C11A—H11C	107 (3)
C14—C13—C11	122.0 (2)	C3A—C2A—C1A	121.7 (2)
C14—C13—H13	121.3 (14)	C3A—C2A—H2A	120.9 (17)
C11—C13—H13	116.6 (14)	C1A—C2A—H2A	117.3 (17)
C13—C14—C15	120.0 (2)	C2A—C3A—C1A^i^	120.8 (3)
C13—C14—H14	119.1 (13)	C2A—C3A—H3A	124.8 (16)
C15—C14—H14	120.7 (13)	C1A^i^—C3A—H3A	114.3 (16)
			
O12—Co1—N1—C2	151.59 (17)	C19—C18—N20—C21	0.9 (3)
O28—Co1—N1—C2	65.85 (17)	C17—C18—N20—Co1	−0.4 (3)
N23—Co1—N1—C2	−27.59 (17)	C19—C18—N20—Co1	−178.70 (14)
N20—Co1—N1—C2	−114.40 (17)	O12—Co1—N20—C18	14.51 (17)
O12—Co1—N1—C5	27.17 (16)	N23—Co1—N20—C18	−167.82 (18)
O28—Co1—N1—C5	−58.57 (16)	N6—Co1—N20—C18	100.61 (17)
N23—Co1—N1—C5	−152.01 (16)	N1—Co1—N20—C18	−76.62 (17)
N20—Co1—N1—C5	121.18 (16)	O12—Co1—N20—C21	−165.07 (14)
C5—N1—C2—C3	−36.4 (2)	N23—Co1—N20—C21	12.60 (15)
Co1—N1—C2—C3	−166.02 (16)	N6—Co1—N20—C21	−78.97 (15)
N1—C2—C3—C4	40.4 (3)	N1—Co1—N20—C21	103.80 (15)
C2—C3—C4—C5	−28.2 (3)	C18—N20—C21—C22	147.73 (19)
C2—N1—C5—C4	18.5 (3)	Co1—N20—C21—C22	−32.7 (2)
Co1—N1—C5—C4	149.80 (18)	N20—C21—C22—N23	41.1 (2)
C3—C4—C5—N1	6.4 (3)	C21—C22—N23—C24	153.4 (2)
O12—Co1—N6—C10	63.71 (15)	C21—C22—N23—Co1	−31.6 (2)
O28—Co1—N6—C10	149.42 (15)	O28—Co1—N23—C24	9.06 (18)
N23—Co1—N6—C10	−117.05 (15)	N20—Co1—N23—C24	−174.31 (18)
N20—Co1—N6—C10	−30.16 (16)	N6—Co1—N23—C24	−82.19 (18)
O12—Co1—N6—C7	−59.05 (15)	N1—Co1—N23—C24	96.26 (18)
O28—Co1—N6—C7	26.66 (15)	O28—Co1—N23—C22	−165.47 (14)
N23—Co1—N6—C7	120.19 (15)	N20—Co1—N23—C22	11.16 (15)
N20—Co1—N6—C7	−152.92 (15)	N6—Co1—N23—C22	103.28 (15)
C10—N6—C7—C8	21.1 (2)	N1—Co1—N23—C22	−78.27 (15)
Co1—N6—C7—C8	149.59 (15)	C22—N23—C24—C26	−177.86 (19)
N6—C7—C8—C9	2.1 (3)	Co1—N23—C24—C26	8.0 (3)
C7—C8—C9—C10	−24.0 (3)	C22—N23—C24—C25	4.1 (3)
C7—N6—C10—C9	−36.7 (2)	Co1—N23—C24—C25	−169.96 (16)
Co1—N6—C10—C9	−165.17 (14)	N23—C24—C26—C27	−16.8 (3)
C8—C9—C10—N6	37.7 (2)	C25—C24—C26—C27	161.2 (2)
C13—C11—O12—Co1	−166.03 (14)	N23—C24—C26—C32	163.0 (2)
C17—C11—O12—Co1	14.8 (3)	C25—C24—C26—C32	−19.0 (3)
O28—Co1—O12—C11	155.40 (15)	C32—C26—C27—O28	−178.51 (19)
N20—Co1—O12—C11	−21.22 (16)	C24—C26—C27—O28	1.3 (3)
N6—Co1—O12—C11	−113.16 (15)	C32—C26—C27—C29	3.4 (3)
N1—Co1—O12—C11	68.36 (16)	C24—C26—C27—C29	−176.76 (19)
O12—C11—C13—C14	178.56 (19)	C29—C27—O28—Co1	−160.24 (14)
C17—C11—C13—C14	−2.2 (3)	C26—C27—O28—Co1	21.7 (3)
C11—C13—C14—C15	−1.2 (3)	O12—Co1—O28—C27	154.22 (16)
C13—C14—C15—C16	2.4 (3)	N23—Co1—O28—C27	−23.40 (16)
C14—C15—C16—C17	−0.3 (3)	N6—Co1—O28—C27	68.36 (16)
C15—C16—C17—C11	−3.1 (3)	N1—Co1—O28—C27	−114.40 (16)
C15—C16—C17—C18	174.49 (19)	O28—C27—C29—C30	179.3 (2)
O12—C11—C17—C16	−176.63 (18)	C26—C27—C29—C30	−2.4 (3)
C13—C11—C17—C16	4.2 (3)	C27—C29—C30—C31	0.0 (4)
O12—C11—C17—C18	5.9 (3)	C29—C30—C31—C32	1.3 (4)
C13—C11—C17—C18	−173.29 (18)	C30—C31—C32—C26	−0.2 (4)
C16—C17—C18—N20	169.29 (18)	C27—C26—C32—C31	−2.2 (3)
C11—C17—C18—N20	−13.3 (3)	C24—C26—C32—C31	178.0 (2)
C16—C17—C18—C19	−12.4 (3)	C3A^i^—C1A—C2A—C3A	0.9 (4)
C11—C17—C18—C19	165.03 (18)	C11A—C1A—C2A—C3A	−179.5 (2)
C17—C18—N20—C21	179.12 (18)	C1A—C2A—C3A—C1A^i^	−0.9 (4)

Symmetry codes: (i) −*x*+2, −*y*, −*z*+1.

### Hydrogen-bond geometry (Å, °)

**Table:**

CgA, CgB and CgC are the centroids of the C11-C17, C26–C32 and C1A–C3A,C1A'–C3A' rings, respectively.

**Table:**

*D*—H···*A*	*D*—H	H···*A*	*D*···*A*	*D*—H···*A*
N1—H1···O4A^ii^	0.90 (2)	2.32 (2)	3.200 (2)	166.2 (19)
N6—H6···O2A	0.89 (2)	2.55 (2)	3.244 (2)	135.9 (18)
N6—H6···O3A	0.89 (2)	2.33 (2)	3.181 (3)	160.9 (18)
C2A—H2A···O2A	0.88 (2)	2.61 (3)	3.477 (3)	166 (2)
C19—H19B···CgA^iii^	0.92 (2)	2.85 (2)	3.438 (2)	122.8 (16)
C14—H14···CgB^iv^	0.94 (2)	2.56 (2)	3.430 (2)	153.8 (17)
C22—H222···CgC^v^	1.00 (2)	2.92 (2)	3.784 (2)	145.5 (16)

Symmetry codes: (ii) *x*−1/2, −*y*+1/2, *z*−1/2; (iii) −*x*+1, −*y*, −*z*; (iv) −*x*+1/2, *y*−1/2, −*z*+1/2; (v) −*x*+3/2, *y*+1/2, −*z*+1/2.
